# Persistent dysbiosis of intestinal and oral microbiota after neoadjuvant radiotherapy for rectal cancer: Implications for surgery and microbiome intervention

**DOI:** 10.3389/fonc.2026.1786044

**Published:** 2026-05-25

**Authors:** Yihao Zhang, Junze Xue, Hao Lin, Kun Wang, Xiaojie Tan, Xuelong Jiao, Haitao Jiang

**Affiliations:** Department of Gastrointestinal Surgery, Affiliated Hospital of Qingdao University, Qingdao, China

**Keywords:** 16S rRNA gene sequencing, intestinal microbiota, neoadjuvant radiotherapy, oral microbiota, rectal cancer

## Abstract

**Background:**

Neoadjuvant radiotherapy represents a conventional approach for managing locally advanced rectal carcinoma. Although this regimen has many benefits, radiation injury is associated with intestinal mucosal injury and gut microbiota dysbiosis. The long-term dynamics of inflammation and microbial recovery, including oral microbiota alterations, remain unclear.

**Methods:**

Intestinal mucosal specimens were obtained from patients with rectal cancer who underwent surgical treatment without radiotherapy or at predetermined time intervals (4 ± 1 weeks, 8 ± 1 weeks and 12 ± 1 weeks) after radiotherapy. The levels of TNF-α, IL-4 and IL-6 in the intestinal mucosa were determined using ELISA. Meanwhile, the intestinal mucosa and saliva of 18 recruited rectal cancer patients were subjected to 16S rRNA gene sequencing (radiotherapy group, n=9; non-radiotherapy group, n=9) for analysis of the gut and oral microbial communities.

**Results:**

Mucosal IL-4 levels were still significantly elevated at 12 weeks post-radiotherapy with respect to the non-radiotherapy group (p<0.05), while TNF-α and IL-6 were back at baseline levels. The gut microbiota composition of radiotherapy patients differed from that of non-radiotherapy patients, with a significant reduction in Chao1 diversity index (p<0.01) and distinct β-diversity clustering (ANOSIM R = 0.614, p=0.001). These differences were still present at 12 weeks post radiotherapy. The oral microbiota changes were limited, with only a few taxa that showed significant differences (ANOSIM R = 0.38, p=0.009).

**Conclusion:**

Neoadjuvant radiotherapy is associated with persistent intestinal mucosa inflammation and prolonged disruption of gut microbiota and this dysbiosis does not fully normalize within 12 weeks after treatment. Changes in oral microbiota appeared less pronounced. These findings provide the biological context for understanding intestinal recovery after radiotherapy and indicate that more studies are needed to direct future efforts towards the peri-operative manipulation of gut microbiota.

## Introduction

Colorectal Cancer (CRC) ranks as the third most frequently diagnosed malignancy worldwide and represents the second leading contributor to cancer-related mortality globally (1). In cases of locally advanced rectal tumors, the standard therapeutic approach involves preoperative radiotherapy combined with subsequent surgical removal to enhance regional tumor management. Although this multimodal treatment strategy has great advantages in oncology, damage to the normal gut wall during radiation treatment still remains a clinically relevant concern. There is accumulating evidence, both in animal models and in clinical studies, that radiotherapy changes the composition and the functions of the gut microbiota (2–4). The radiation-induced changes of the microbiota are thought to be involved in the pathogenesis of the radiation enteritis and might affect the treatment (4).

The intestinal epithelium is very sensitive to radiation damage due to the high rate of cell renewal (5). While radiotherapy targets tumor cells, it also affects normal tissues and the gut microbiota. The high-energy rays of radiotherapy inevitably damage healthy cells, thus causing certain radiotoxicity (6). Specifically, the ionizing radiation from radiotherapy could damage the integrity of the epithelium thus leading to an inflammatory response and impaired intestinal mucosal barrier function, ultimately resulting in radiation enteritis. Research conducted by Hauer-Jensen demonstrated that nearly 90% of individuals undergoing pelvic or abdominal radiotherapy report digestive disturbances during the initial treatment phase (7). While the clinical symptoms can be improved with time, there is growing evidence indicating that subclinical intestinal injury might remain for a long period after radiotherapy ends.

Gut microbes themselves or their derived products like SCFAs, tryptophan-derived metabolites can regulate the metabolism and immunity of the host (8). More and more studies show that gut microbiota dysbiosis is closely associated with the occurrence of some intestinal diseases and even cancers. Additionally, the metabolites from the gut microbiota also play important roles in cancers or tumor related diseases (9, 10). Radiotherapy changes the composition of the gut microbiota. In preclinical studies it has been shown that ionizing radiation decreases the microbial diversity and changes the relative abundance of the phyla such as *Firmicutes* and *Proteobacteria* (2, 4). These dysbiotic changes have been found in patients receiving pelvic radiotherapy and were associated with the gastrointestinal symptoms and the mucosal injury (11). However the temporal pattern of the microbiota recovery after radio therapy, in particular during the clinically relevant of 8–12 weeks waiting period before the surgery, is still poorly characterized.

Surgery after neoadjuvant radiotherapy has usually been decided according to the tumor response and technical considerations, with an intermission of at least 8–12 weeks, but limited data are available regarding the status of intestinal inflammation and microbiota recovery during this period. Understanding more about those processes might allow additional justification for perioperative decisions as well as research into adjuncts that can accelerate intestinal recovery.

Meanwhile, there are literature reports indicating that the oral microbiota of CRC patients also undergoes corresponding changes. Compared with the healthy control group, there are significant differences in the bacteria present in the oral rinse samples of CRC patients (12).

As an integral part of the digestive tract microbial community, the oral microbiota has a certain connection with the intestinal microbiota. Many studies have revealed that the feces of patients with CRC or adenomas are rich in oral-type bacteria (13–15). *Fusobacterium nucleatum* is an opportunistic symbiotic anaerobic bacterium present in the oral cavity, which can cause periodontal disease (16). It can also trigger inflammation and tumorigenesis, thereby promoting the development and progression of CRC (17, 18). Analyzing oral-related bacteria may be valuable for the detection of CRC (19); thus, the impact of oral dysbiosis on CRC cannot be ignored.

In the present work, we investigated inflammatory cytokine profiles and intestinal microbial composition of rectal cancer patients at different time points after radiotherapy. Also, we did a preliminary exploration on the oral microbiomes to assess concurrent microbial changes. With inflammation information and microbiome data, this study sought to characterize post-radiotherapy intestinal recovery and to provide preliminary biological insights relevant to clinical management.

## Material and methods

### Study design and ethics approval

This study was a cross-sectional observational study that was designed to study the status of intestinal mucosal inflammation and the composition of the intestinal and oral microbiota of the rectal cancer at different time points after the neoadjuvant radiotherapy. The research involved collecting both gut mucosal specimens and salivary samples from individuals diagnosed with rectal carcinoma. These participants were divided into two groups: one undergoing immediate surgical intervention without prior radiation treatment and another receiving surgery 4–12 weeks post-radiotherapy. The levels of inflammatory factors (TNF, IL-4 and IL-6) in the samples were measured. In addition, using high throughput sequencing technology, we also analyzed the 16S rRNA of bacteria from rectal sample and saliva sample in 18 rectal cancer patients who had received or not received radiotherapy, aim to explore the change of intestinal flora and oral microbiota after receiving radiotherapy and to assess recovery from radiation enteritis.

**Figure 1:**
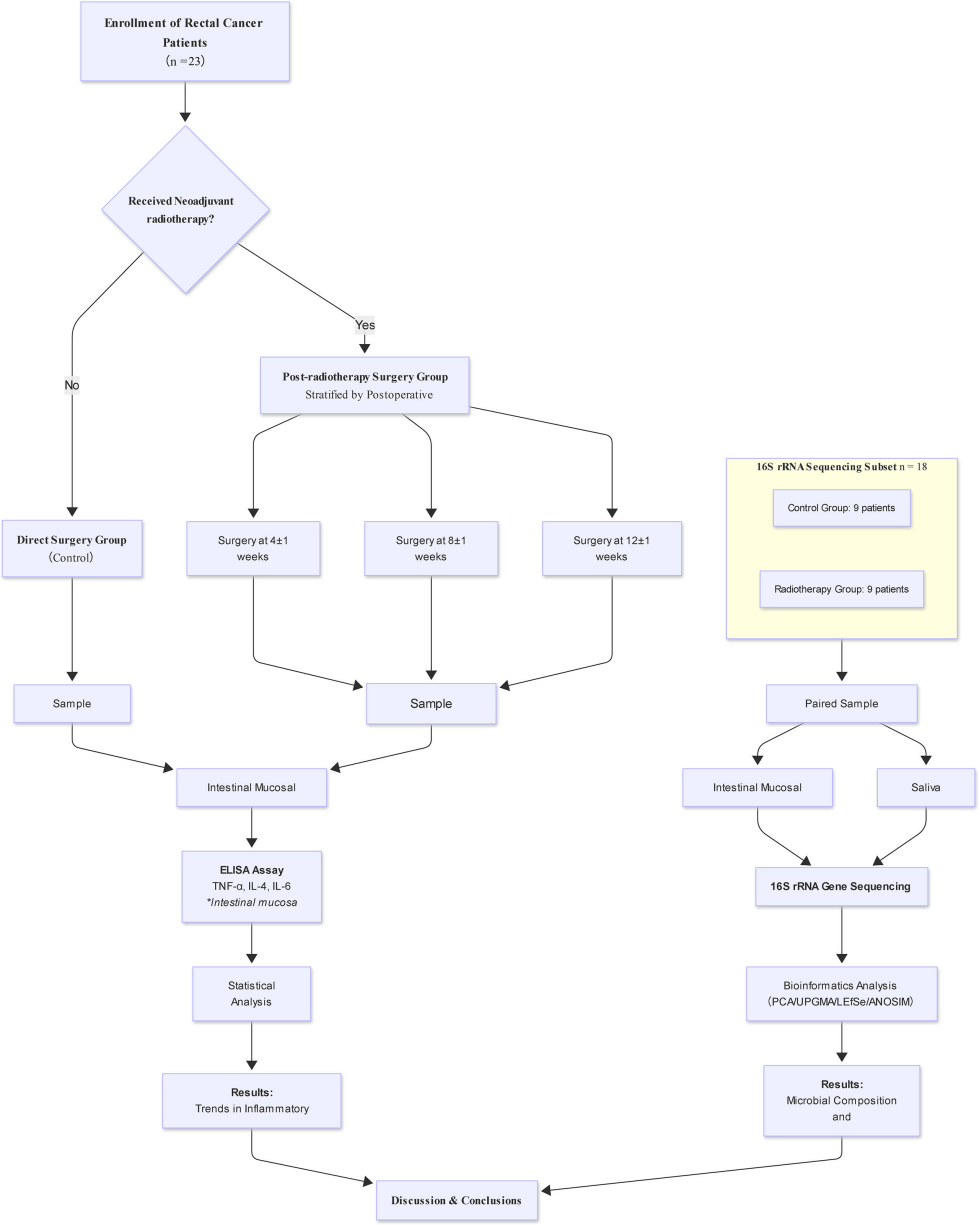
Flowchart of study design and analytical pipeline for assessing intestinal and oral microbiota changes following neoadjuvant radiotherapy in rectal cancer patients.

The human-related studies were approved by the Medical Ethics Committee of Qingdao University Affiliated Hospital (QYFYEC 2024-237). The overall study flow is shown in **Figure 1**.

### Study population

Intestinal (rectal) mucosal tissues were obtained from patients diagnosed with rectal cancer who underwent surgical resection at our hospital. There are total twenty-three patients included in the study. These samples were categorized into four groups based on their clinical treatment pathway, which was determined by tumor stage, resectability, and patient preference following multidisciplinary team discussion: (1) no radiotherapy (control, n=9 for inflammatory analysis), (2) 4-week subgroup (RT-4w): Surgery performed at 4 ± 1 weeks post-radiotherapy (n=5 for inflammatory analysis), (3) 8-week subgroup (RT-8w): Surgery performed at 8 ± 1 weeks post-radiotherapy (n=5 for inflammatory analysis) and (4) 12-week subgroup (RT-12w): Surgery performed at 12 ± 1 weeks post-radiotherapy (n=4 for inflammatory analysis).

Inclusion criteria were: (1) age ≥ 18 years, (2) histologically confirmed rectal adenocarcinoma, (3) Eastern Cooperative Oncology Group (ECOG) performance status 0–2, and (4) availability of intestinal mucosal tissue samples during surgery.

Exclusion criteria were: (1) documented inflammatory bowel disease (e.g., Crohn’s disease, ulcerative colitis); (2) administration of intravenous chemotherapeutic agents, molecularly targeted therapies, immunosuppressive medications, corticosteroids, or antimicrobial drugs within 14 days prior to surgery (oral capecitabine was permitted as part of standard neoadjuvant chemoradiotherapy); (3) history of fecal diversion procedures; (4) concurrent active infections requiring systemic antibiotic therapy; and (5) pregnancy or lactation.

### Sample collection

Intestinal mucosal samples: intestinal mucosal samples: Full-thickness or mucosal biopsy samples from the rectal wall at least 5 cm proximal to the tumor margin were collected during surgery in order to avoid a direct contamination of the samples by the tumor. The samples were immediately rinsed three times with ice-cold phosphate-buffered saline (PBS, pH 7.4) to wash out the residual blood and the content of the lumen. After rinsing, samples were snap-frozen in liquid nitrogen and stored at -80 C. A part of each sample (about 0.2 g) was used for the measurement of the inflammatory cytokines by ELISA, and an adjacent part of the sample for the extraction of DNA and the 16S rRNA gene sequencing.

Saliva samples: unstimulated whole saliva (approx. 2–3 mL) was collected from each patient on the morning of surgery, before any preoperative fasting or medication administration. Patients were asked to rinse their mouth with water and then passively drool into a sterile collection tube for 5 minutes. Samples were put on ice, aliquoted in cryotubes and stored at -80 C until DNA extraction. Samples with visible blood contamination (e.g., due to gingival bleeding) or insufficient volume (< 0.5 mL) were excluded from downstream microbiome analysis.

### Radiotherapy regimen

All patients of the neoadjuvant radiotherapy group got intensity-modulated radiotherapy (IMRT). Total dose was prescribed 50-50.4 Gy, in 25–28 fractions of 1.8-2.0 Gy per fraction, five times in a week. The clinical target volume was the primary tumor and the mesorectal lymph nodes.

### Levels of TNF-α, IL-4 and IL-6 were determined using an ELISA assay

Intestinal mucosal tissue samples (0.2 g) were collected and rinsed three times with phosphate-buffered saline (PBS), maintaining all procedures at 4 °C. The extraction solution was prepared by combining cell disruption buffer with protease inhibitor at a 100:1 volume ratio, then was mixed thoroughly using a vortex mixer. The washed tissues were transferred into a 2.5-ml microcentrifuge tube, followed by the addition of 1 ml of the prepared lysis solution together with steel beads for disruption. The tissues were homogenized with an ultrasound disruptor; after that, the homogenate was centrifuged at 3,000 rpm for 15 minutes. Then, the supernatant was obtained for further studies. The inflammatory cytokines (TNF-α, IL-4 and IL-6) were quantified using commercial ELISA kits following the protocols provided by the manufacturer (Jingmei Biotech, Jiangsu, China).

### 16S rRNA gene sequencing and bioinformatics

Genomic DNA of the samples was extracted using a bacterial genomic extraction kit (DP302-02, TIANGEN) and the DNA was quantified with Qubit (Invitrogen, USA). Specific oligonucleotide primers were selected based on targeted sequencing regions.

For all samples in this study, the V3-V4 hypervariable region of the 16S rRNA gene was amplified using the primer pair 341F (5’-CCTACGGGNGGCWGCAG-3’) and 805R (5’-GACTACHVGGGTATCTAATCC-3’).

Amplified DNA fragments were cleaned using AMPure XT magnetic beads (Beckman Coulter Genomics, Danvers, MA, USA) and their concentrations were reassessed with the Qubit system (Invitrogen, USA).

The sequencing libraries were constructed by using the NEBNext Ultra II DNA Library Prep Kit (NEB, USA) according to the manufacturer protocol. The paired-end sequencing (2 X 250 bp) was carried out on the NovaSeq 6000 platform (Illumina, USA).

Raw paired-end sequencing data (RawData) were first merged based on overlapping regions. Low-quality sequences and chimeras were then filtered out to obtain high-quality CleanData. Denoising was performed using the DADA2 pipeline within QIIME2 to correct PCR amplification and sequencing errors, generating Amplicon Sequence Variants (ASVs) and an ASV abundance table. Subsequent analyses included alpha diversity (Chao1), beta diversity (Bray-Curtis, ANOSIM), taxonomic composition (phylum/genus level, LEfSe with LDA > 3.0).

### Statistical analysis

The data analysis was conducted utilizing SPSS version 26.0. For normally distributed quantitative variables, results were presented as averages accompanied by standard deviation values. The Mann-Whitney U test was used to compare the non-radiotherapy group and the radiotherapy group. One-way analysis of variance (ANOVA) was employed to compare the differences among groups at different time points after radiotherapy. A p-value < 0.05 was considered statistically significant. The notations for significant differences were as follows: *p < 0.05; **p < 0.01.

### Sample size consideration

Because of the pilot and exploratory nature of this study, we did not do a formal *a priori* sample size calculation. A *post-hoc* power analysis for the main outcome (microbial beta-diversity, ANOSIM) shows that with the current sample size (n=9 per group) we have 80% power to detect a large effect size (R > 0.5) at alpha = 0.05. Findings should be seen as hypothesis generating and needs to be validated in larger cohorts.

## Results

### Significant changes in inflammatory factors produced by intestinal mucosa after radiotherapy

**Figure 2:**
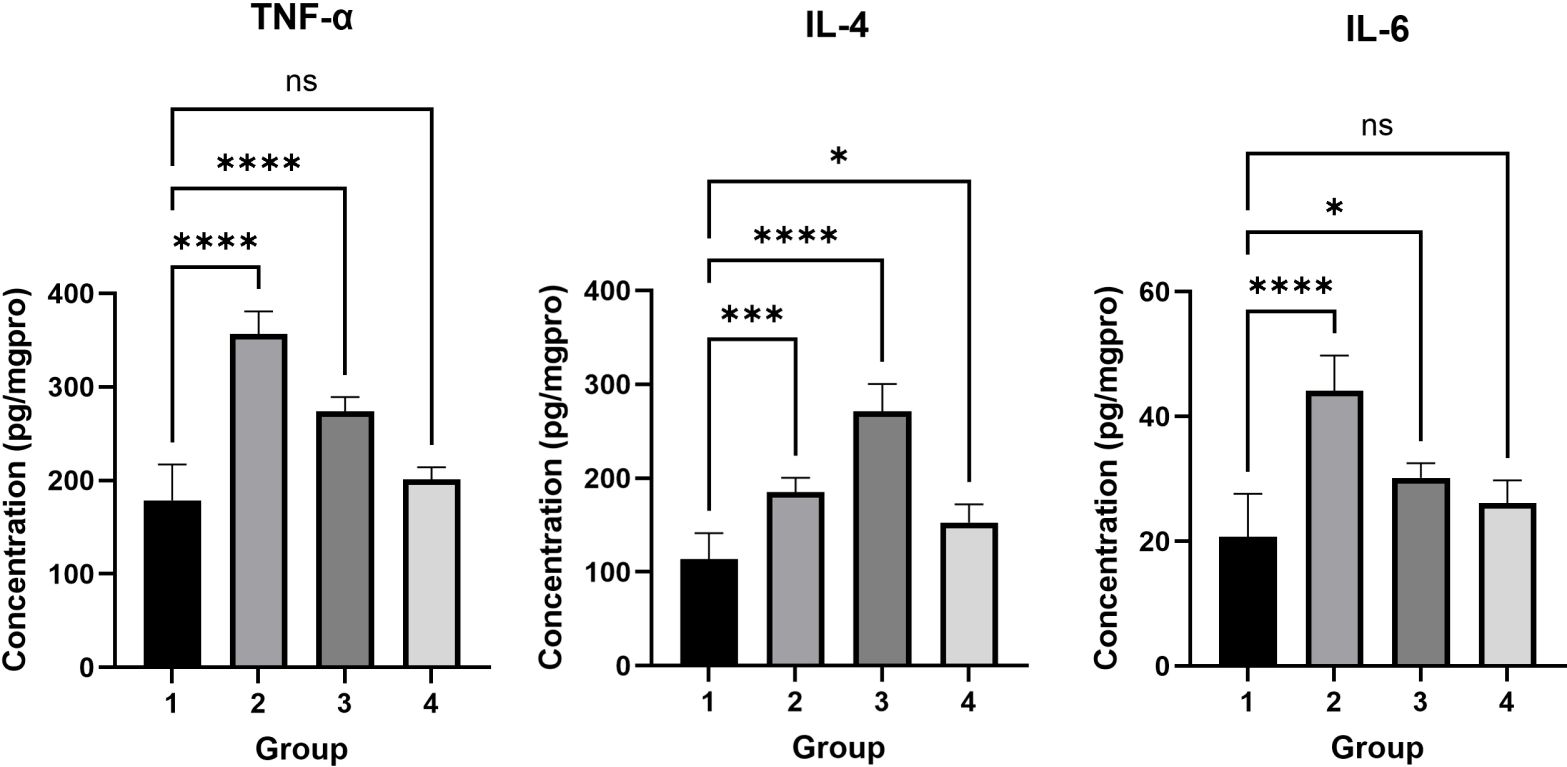
Cytokine levels in intestinal mucosa after neoadjuvant radiotherapy. Mucosal concentrations of TNF-α, IL-4 and IL-6 were measured by ELISA in patients who did not receive radiotherapy (Group 1, n=9) and in patients at 4 ± 1 weeks (Group 2, n=5), 8 ± 1 weeks (Group 3, n=5) and 12 ± 1 weeks (Group 4, n=4) after radiotherapy completion. *Data are expressed as mean ± SD. Statistical analysis was performed using one-way ANOVA followed by a *post hoc* test. Significance is indicated as *p < 0.05, ***p < 0.001 and ****p < 0.0001, compared with the no-radiotherapy group (Group 1).

Neoadjuvant radiotherapy significantly increased the mucosal levels of the pro-inflammatory cytokines TNF-α and IL-6 compared to the non-radiotherapy group (**Figure 2**). After 12 weeks, both cytokines returned to baseline levels, showing no significant difference from the non-radiotherapy group (ns). In contrast, the anti-inflammatory cytokine IL-4 exhibited a different pattern. IL-4 levels were significantly elevated at 4 weeks and 8 weeks post-radiotherapy and, importantly, remained substantially higher than those in the non-radiotherapy group at 12 weeks (*p < 0.05) (**Figure 2**).

**Figure 3:**
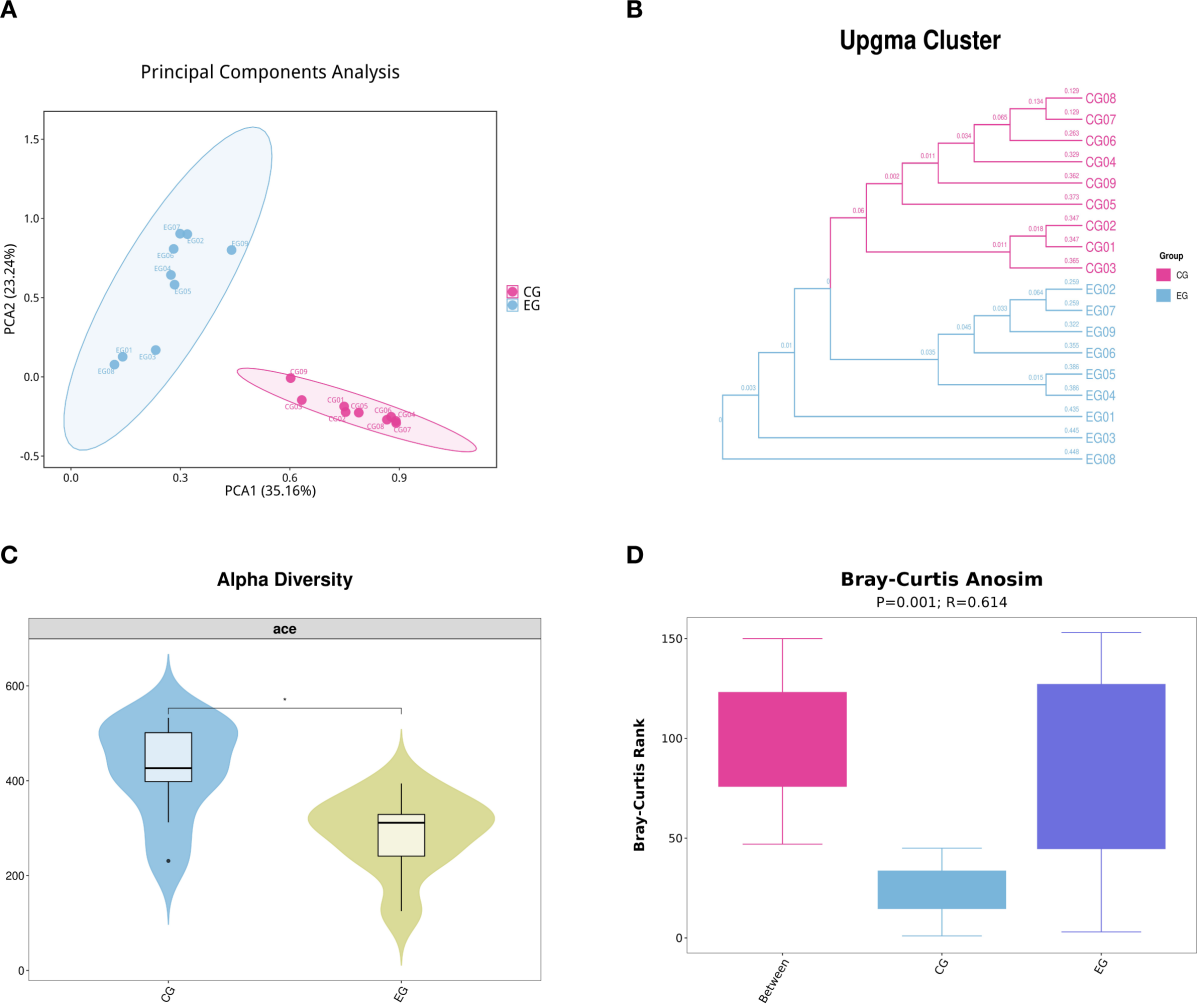
**(A)** presents PCA, **(B)** represents cluster analysis. **(C)** presents the alpha diversity between non-radiotherapy and post-radiotherapy patients. The Chao1 index was significantly lower in the EG group than in the CG group. The results indicate reduced species richness. Error bars represent standard error of the mean (SEM). Statistical significance was calculated using Mann-Whitney U test (*p < 0.05). **(D)** shows the ANOSIM analysis of gut microbiota beta diversity. The differences between two groups (ANOSIM R = 0.614, P = 0.001) suggest strong community-level differentiation caused by radiotherapy.

Intestinal mucosa samples were obtained from 18 patients with rectal cancer, which were divided into two groups: the radiotherapy group (EG, n=9) and the non-radiotherapy group (CG, n=9). As shown in **Figure 3**, 16S rRNA sequencing combined with principal component analysis (PCA) and Unweighted Pair Group Method with Arithmetic Mean (UPGMA) clustering revealed a distinct separation between the two groups, indicating an alteration in the intestinal flora. Alpha diversity, as measured by the Chao1 index, suggests that there was a lower number of microbial species in the neoadjuvant radiotherapy group. Anosim using Bray-Curtis distances demonstrated that the difference between post-radiotherapy (EG) and non-radiotherapy (CG) was greater than the variation within each group (R = 0.614, P = 0.001), suggesting an extensive radiotherapy-mediated shift in microbiota composition.

**Figure 4:**
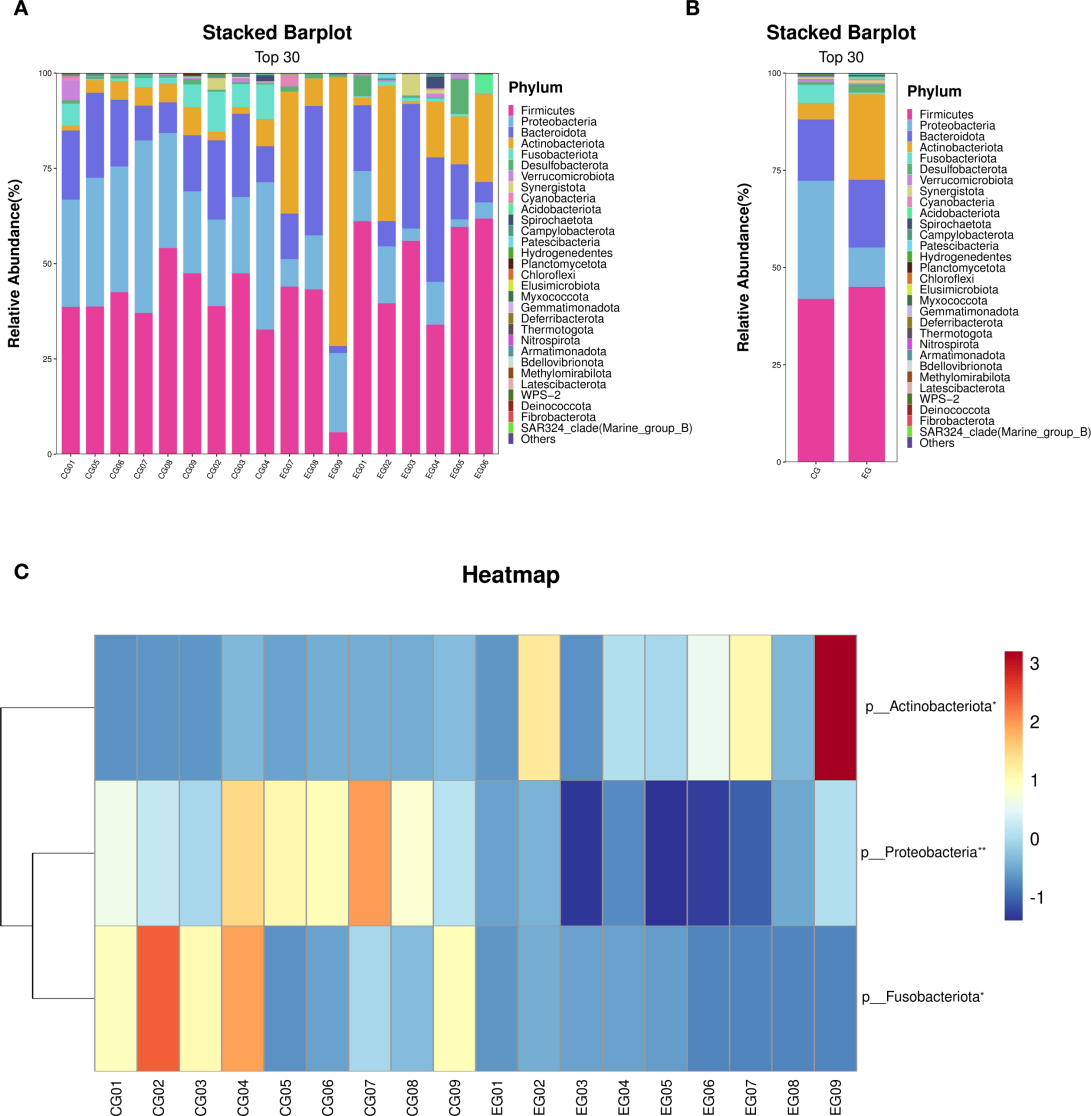
The taxonomic composition analysis results through stacked bar charts, highlighting the 30 most abundant phyla in each specimen. **(A)** a stacked bar chart of the relative abundance of species at the phylum level based on OTU, where the x-axis represents the sample name, the y-axis (Relative Abundance) represents the relative abundance and “Others” represents the sum of the relative abundance of all other phyla besides these 30 phyla in the figure. **(B)** presents a grouped comparison of phylum-level distributions, where the horizontal axis indicates sample categories and the vertical axis shows relative abundance percentages. Similarly, the “Others” designation combines all phyla beyond the 30 most prevalent ones. ANOSIM analysis showed significant differences in similarity between groups (R = 0.6118, p=0.001). **(C)** a Heatmap of differentially abundant bacterial phyla. The heatmap displays Z-score normalized relative abundance of three bacterial phyla that showed significant differences between the radiotherapy (EG) and non-radiotherapy (CG) groups. Bacterial phyla were identified using LEfSe (LDA > 2.0, p < 0.05) and validated by the Mann-Whitney U test with FDR correction (q < 0.05). Rows represent phylum; columns represent individual samples. The color scale indicates Z-score values (red: higher abundance; blue: lower abundance).

**Figure 5:**
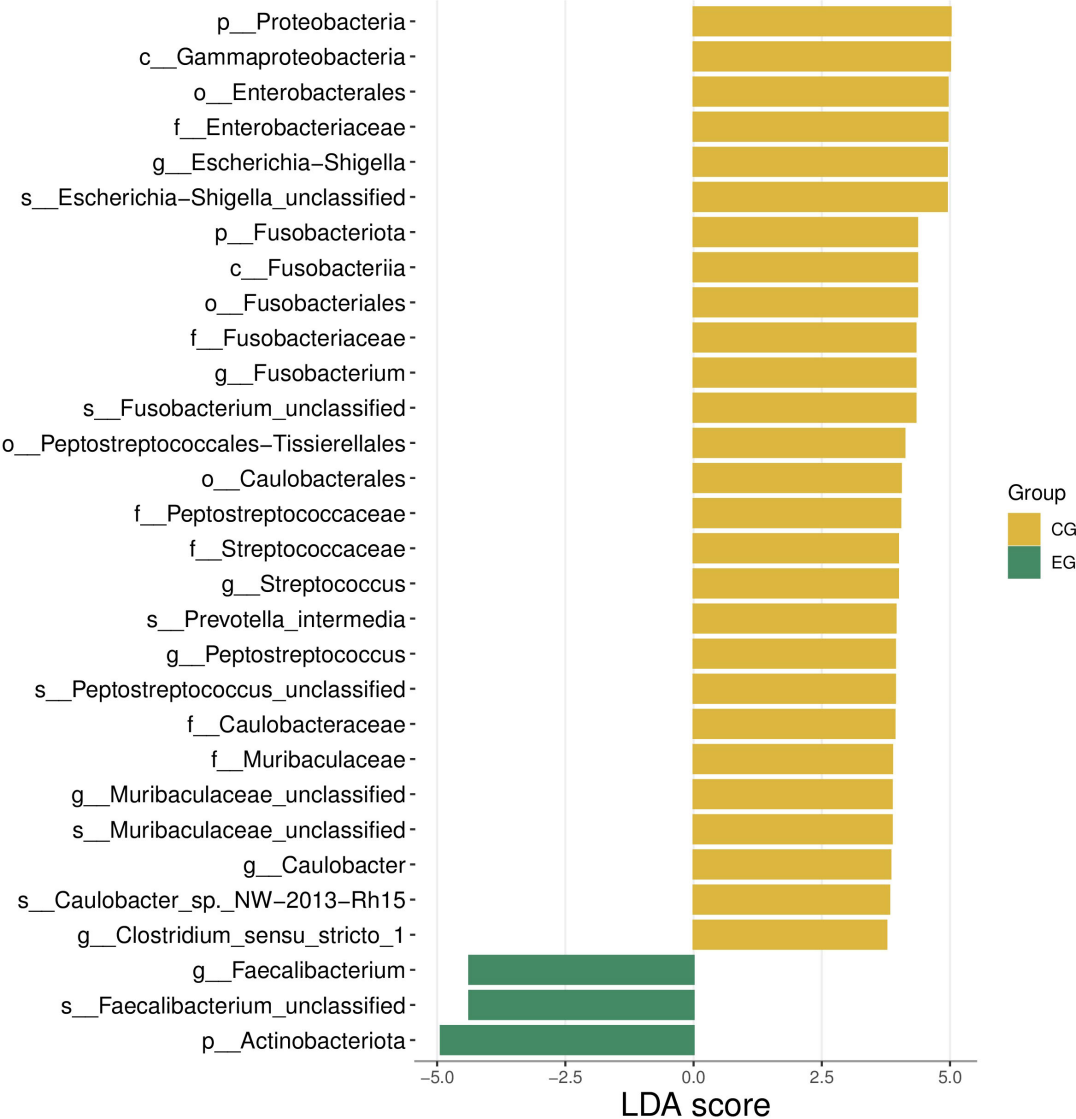
A histogram of LDA value distribution based on OTUs. The histogram displays species with an LDA score greater than the set threshold (default: 3), which are biomarkers with statistical differences between different groups. The histogram shows significant differences in species abundance among different groups and the length of the bars represents the magnitude of the influence of different species (i.e., the LDA score).

Meanwhile, ANOSIM analysis indicated a significant difference (R = 0.6118, p = 0.001) in the top 10 most abundant gut microbial communities at the phylum level between the two groups. **Figure 4** consists of species composition analysis diagrams (**Figures 4A, B**) and a heatmap (**Figure 4C**). The species composition analysis diagram (**Figures 4A**, **B**) illustrate the relative abundance of microbial species at the phylum level based on Operational Taxonomic Units (OTUs). In terms of differences between these two groups, the differences in the *Proteobacteria* and *Fusobacteriota* communities were extremely significant (P< 0.01), while the differences in the *Actinobacteriota* and *Planctomycetota* communities were significant (P< 0.05). **Figure 4C** shows a heatmap of the three bacterial phyla that had differences between the radiotherapy and non-radiotherapy (LEfSe, LDA > 2.0, p < 0.05; Mann-Whitney U test, FDR q < 0.05). The heatmap shows that there are differences of abundances between the two groups, suggesting that the radiotherapy may affect only some taxa rather than inducing a dysbiosis of the whole. More detailed results are in **Figure 5**.

LEfSe (LDA Effect Size) analysis was used to identify statistically significant and biologically relevant taxonomic biomarkers differentiating the groups. **Figure 5** is a histogram of LDA value distribution across operational taxonomic units (OTUs). The analytical results show that in the radiotherapy group, the bacterial groups *Actinobacteriota* and *Faecalibacterium* exhibit notable variations; while in the untreated group, 25 microbial taxa including *Proteobacteria*, *Gammaproteobacteria* and *Enterobacterales* show significant differences.

### As time passes after radiotherapy, the gut microbiota still fail to return to normal levels

**Figure 6:**
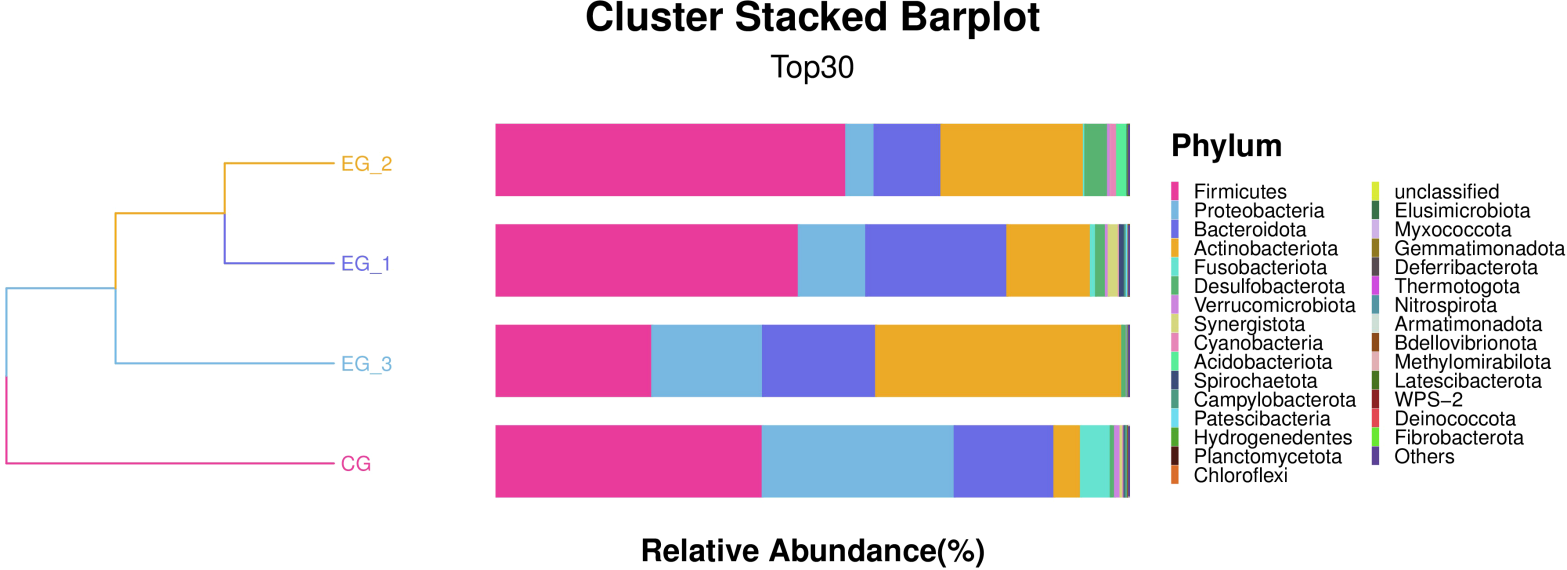
A stacked bar chart of species relative abundance at the phylum level based on OTUs, where the x-axis represents sample names, corresponding to CG (non-radiation group), EG_1 (4 ± 1 weeks post-radiation), EG_2 (8 ± 1 weeks post-radiation) and EG_3 (12 ± 1 weeks post-radiation) respectively. The y-axis (Relative Abundance) indicates the relative abundance. Among them, CG01-CG09 belong to the CG group, EG01-EG04 belong to the EG_1 group, EG05-EG07 belong to the EG_2 group and EG08-EG09 belong to the EG_3 group.

The intestinal samples from 18 patients were divided into the non-radiotherapy group (CG) and groups with different post-radiotherapy time intervals (EG_1, EG_2, EG_3) for comparison. According to the species composition analysis shown in **Figure 6**, it can be observed that even more than 3 months after radiotherapy, the patients’ gut microbiota still differed from the non-radiotherapy group.

### Radiotherapy induces differences in the oral flora of patients

**Figure 7:**
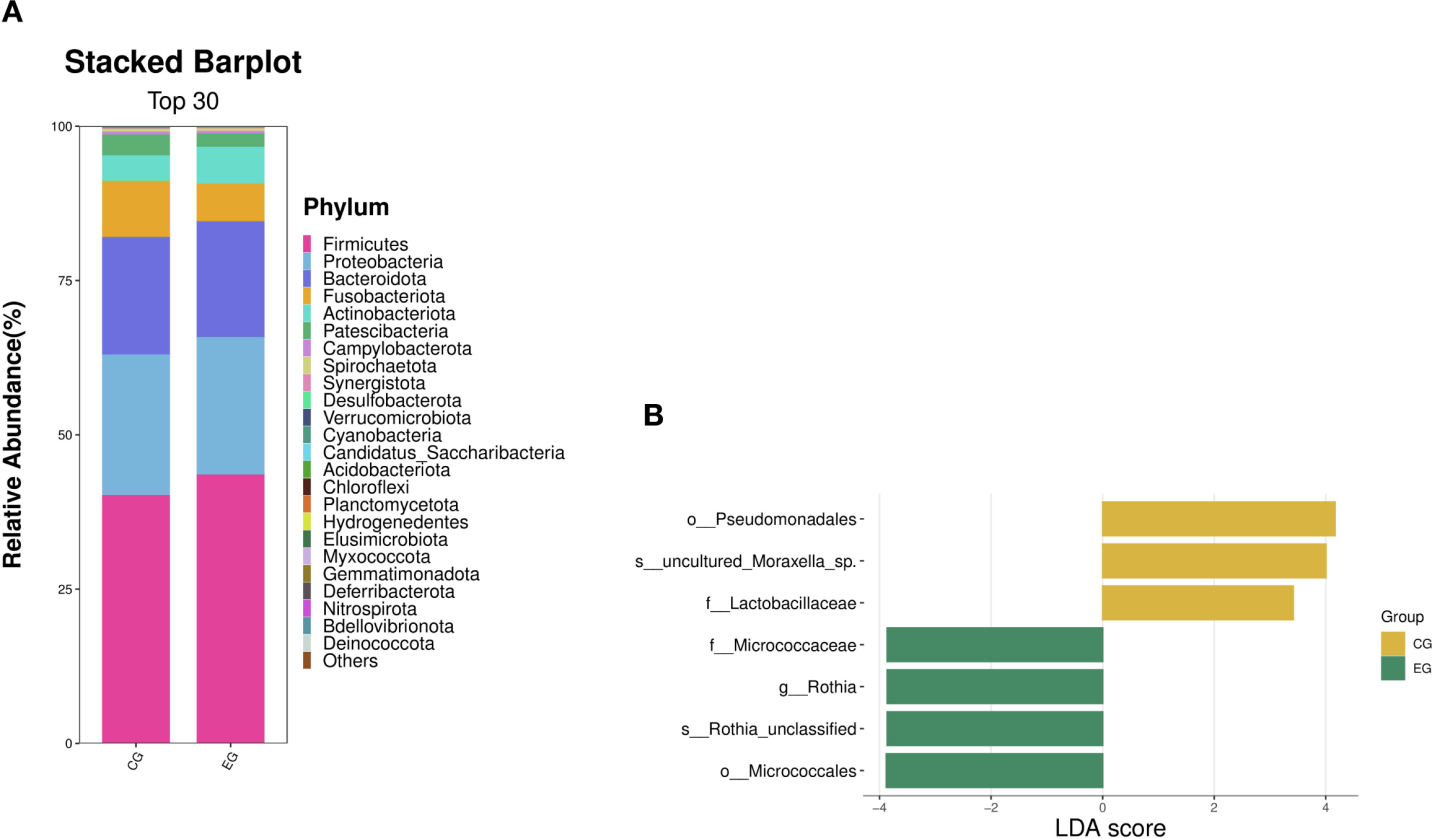
**(A)** a stacked bar chart of the relative abundance of species at the phylum level derived from OTU data. **(B)** a histogram of LDA scores based on OTUs.

In this experiment, saliva specimens were also collected from the same 18 patients with rectal cancer, which were divided into two cohorts: the radiotherapy group (EG, n=9) and the non-radiotherapy group (CG, n=9). Utilizing 16S rRNA gene sequencing technology, notable variations in the top 10 microbial communities at the phylum level between the two groups were observed (ANOSIM analysis: R = 0.38, p=0.0091), as shown in **Figure 7A**. There was an extremely significant difference in the *Candidatus Saccharibacteria* community between the two groups (P<0.05). **Figure 7B** is a histogram of LDA value distribution based on OTUs. The analysis showed that in the radiotherapy group, oral microbial communities such as *Micrococcales* and *Rothia* exhibited significant microbial shifts, while in the non-treatment group (CG), oral microbial communities such as *Pseudomonadales* and *Lactobacillaceae* showed significant differences. Despite some alterations, the changes in the microbiota are highly restricted.

### Correlation analysis between oral microbiota and intestinal microbiota

To explore potential associations between oral and intestinal microbial communities, we also performed Spearman correlation analysis between the relative abundance of the different bacterial phyla in the saliva and the relative abundance of the same phyla in the intestinal mucosal of the same 18 patients.

**Figure 8:**
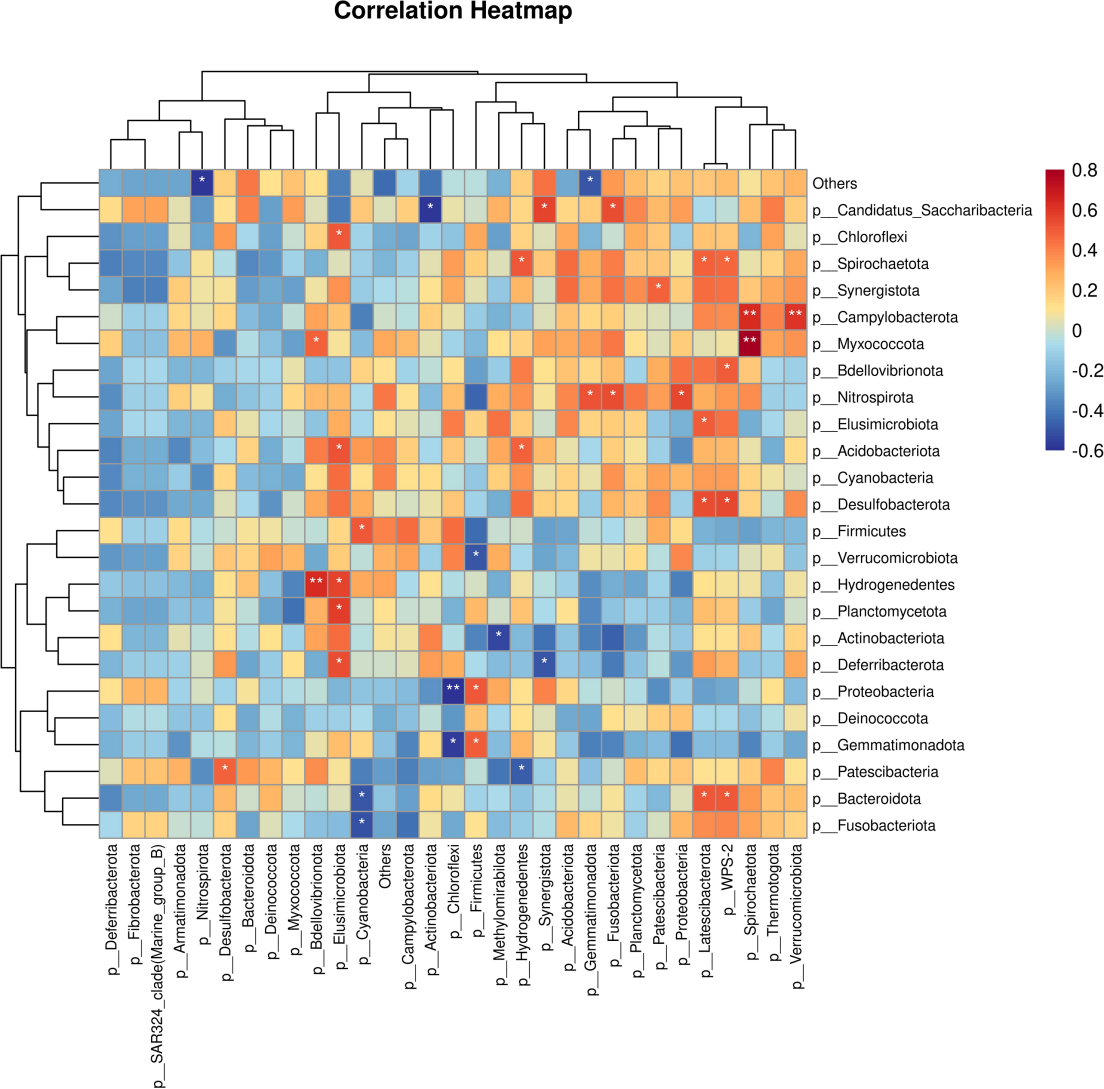
Spearman correlation heatmap between oral and intestinal microbiota at the phylum level. The heatmap displays Spearman rank correlation coefficients (ρ) between the relative abundance of bacterial phyla in oral saliva (x-axis) and intestinal mucosa (y-axis) from paired samples of 18 rectal cancer patients. Red indicates positive correlation; blue indicates negative correlation. Only statistically significant correlations are marked (*p < 0.05, **p < 0.01).

As shown in **Figure 8**, multiple significant positive and negative correlations were observed between oral and intestinal phyla. For example, the abundance of oral Firmicutes showed a moderate positive correlation with intestinal Proteobacteria (ρ≈ 0.5, p<0.05), while oral Bacteroidota exhibited a negative correlation trend with intestinal Actinobacteria (ρ≈-0.4). Several correlations remained statistically significant after FDR correction (q<0.05), suggesting a degree of coordinated variation between the oral and gut microbiota.

However, the majority of correlation coefficients between oral and intestinal phyla had absolute values below 0.4 and did not reach statistical significance, indicating that the overall strength of association between the two compartments is limited.

## Discussion

Radiation-induced intestinal injury is a frequent issue during the treatment of rectal cancer. The intestinal epithelium undergoes rapid renewal. This characteristic increases its sensitivity to ionizing radiation. For this reason, gastrointestinal injury occurs in a considerable proportion of patients after radiotherapy, either shortly after treatment or at later stages (7, 20). In the present study, we examined inflammatory cytokine profiles together with intestinal and oral microbiota in rectal cancer patients after receiving neoadjuvant radiotherapy. Our main findings are the following: ① mucosal IL-4 levels showed a transient increase at 4–8 weeks post-radiotherapy and remained elevated at 12 weeks, whereas TNF-α and IL-6 returned to baseline by 12 weeks; ② gut microbiota composition differed significantly between radiotherapy and non-radiotherapy patients, with reduced alpha diversity and distinct beta-diversity clustering that persisted beyond 12 weeks; ③ changes in oral microbiota were also detected, although their extent was smaller, and there are some correlations in oral-gut microbial.

### Inflammatory responses after radiotherapy

In this study, patients treated with radiotherapy exhibited distinct patterns of mucosal cytokine changes. TNF-α and IL-6, both cytokines are closely involved in intestinal inflammation and epithelial barrier injury. Previous studies demonstrated their effects on tight junction integrity and intestinal permeability (21–23). Those cytokines were elevated at early time points but returned to baseline levels by 12 weeks post-radiotherapy in our study. This suggests that the acute pro-inflammatory response may subside within 8–12 weeks after the radiotherapy. IL-4 followed a different pattern. IL-4 is involved in immunity and Th1/Th2 balance (24–26). IL-4 levels increased at 4 and 8 weeks and remained significantly elevated at 12 weeks compared to the non-radiotherapy group. This prolonged elevation of IL-4 may represent a sustained anti-inflammatory or tissue-repair response.

### Gut microbiota changes persist after radiotherapy

The gut microbiota analysis revealed clear and sustained differences between patients with and without radiotherapy. Multivariate methods (PCA, UPGMA, ANOSIM) consistently separated the two groups, with a large effect size (R = 0.614, p=0.001). The Chao1 diversity index was significantly lower in the radiotherapy group, indicating reduced species richness. This separation was observed across samples. Ionizing radiation is known to interfere with host–microbe interaction and it also change microbial metabolite and damage the epithelial barrier function (6, 27). Moreover, when patients were separated according to their post-radiotherapy time period, the gut microbiota profile did not resemble that of the non-radiotherapy group. This altered pattern persisted more than three months after radiotherapy.

### Oral microbiota and oral-gut axis

Previous reports described potential links between oral and intestinal microbiota in colorectal cancer and radiotherapy response (28). In this work, we observed differences in oral microbial composition between radiotherapy and non-radiotherapy groups, particularly in taxa such as *Candidatus Saccharibacteria*, *Micrococcales*, and *Rothia.*

However, these changes were less pronounced than those observed in the gut, with ANOSIM R = 0.38 (p = 0.009) and only limited taxa showing significant differences. To explore potential links between oral and intestinal microbial communities, we performed Spearman correlation analysis at the phylum level (**Figure 8**). While several significant correlations were observed (oral Firmicutes and intestinal Proteobacteria), the majority of correlation coefficients had absolute values below 0.4 and did not reach statistical significance, indicating that the overall strength of association between the two compartments is limited. These findings should be considered exploratory, and further studies with larger cohorts are warranted to validate the oral–gut microbial axis in the context of radiotherapy.

### Clinical implications

Surgical resection is commonly recommended by clinical guidelines between 8 and 12 weeks following the completion of neoadjuvant radiotherapy (29). The present study does not challenge this recommendation. Instead, it provides descriptive biological observations. Intestinal inflammation and microbial imbalance were detectable during this interval. The clinical relevance of these findings remains uncertain. Further studies are needed to clarify whether such changes influence postoperative outcomes.

Several strategies have been proposed to reduce radiation-related intestinal injury. These strategies include the use of probiotics and fecal microbiota transplantation. Prior studies reported beneficial effects of bacteria such as *Lactobacillus*, *Bifidobacterium*, *Faecalibacterium prausnitzii* and *Akkermansia muciniphila* on intestinal inflammation and barrier function (20, 30). Fecal microbiota transplantation has also been applied to restore microbial diversity in gastrointestinal diseases (31). Our findings provide biological context for such interventions by characterizing the persistent inflammatory and microbial alterations during the preoperative waiting period.

### Limitations

Several limitations should be considered. First, the sample size was limited, particularly after stratification by post-radiotherapy interval. A *post-hoc* power analysis indicated that the study was adequately powered to detect large effect sizes but may be underpowered for small-to-moderate effects. Second, a cross-sectional design was used. Different patients were compared at different time points and individual longitudinal changes were not assessed. For this reason, the findings should not be interpreted as evidence of recovery within the same individuals. Third, functional metabolite analyses were not performed. Fourth, direct correlations between oral and intestinal microbiota were limited, and the oral-gut axis findings should be considered exploratory. Fifth, we did not assess clinical endpoints, such as postoperative complications or long term recovery. Future longitudinal studies with larger cohorts and integration of clinical endpoints are needed to validate and extend these observations.

## Conclusion

This study examined intestinal and oral inflammatory and microbial features in rectal cancer patients after neoadjuvant radiotherapy. Intestinal inflammatory markers (sustained IL-4 elevation) and gut microbiota composition differed from those observed in patients without radiotherapy and these differences were still detectable up to 12 weeks after treatment. Changes in oral microbiota were present but less pronounced. Oral-gut microbial correlations were present but limited. These findings provide descriptive biological insights into post-radiotherapy intestinal recovery and support the need for future longitudinal and mechanistic studies to inform perioperative microbiota-targeted interventions.

## Data Availability

The raw data supporting the conclusions of this article will be made available by the authors, without undue reservation.
