# Supplementary material for: Persistent dysbiosis of intestinal and oral microbiota after neoadjuvant radiotherapy for rectal cancer: Implications for surgery and microbiome intervention
Source: Front Oncol. 2026 May 25;16:1786044. doi: 10.3389/fonc.2026.1786044 (PMC13243090; doi:10.3389/fonc.2026.1786044)
Supplement: Supplementary file 1 [file DataSheet1.pdf]

Study Cohort and Sample Allocation

Patient ID	Group	Radiotherapy	Time point post-RT (weeks)	Inflammatory markers	Microbiome analysis	Intestinal sample	Saliva sample
CG01	Control	No	-	Yes	Yes	Yes	Yes
CG02	Control	No	-	Yes	Yes	Yes	Yes
CG03	Control	No	-	Yes	Yes	Yes	Yes
CG04	Control	No	-	Yes	Yes	Yes	Yes
CG05	Control	No	-	Yes	Yes	Yes	Yes
CG06	Control	No	-	Yes	Yes	Yes	Yes
CG07	Control	No	-	Yes	Yes	Yes	Yes
CG08	Control	No	-	Yes	Yes	Yes	Yes
CG09	Control	No	-	Yes	Yes	Yes	Yes
EG01	RT-4w	Yes	4±1	Yes	Yes	Yes	Yes
EG02	RT-4w	Yes	4±1	Yes	Yes	Yes	Yes
EG03	RT-4w	Yes	4±1	Yes	Yes	Yes	Yes
EG04	RT-4w	Yes	4±1	Yes	Yes	Yes	Yes
EG05	RT-8w	Yes	8±1	Yes	Yes	Yes	Yes
EG06	RT-8w	Yes	8±1	Yes	Yes	Yes	Yes
EG07	RT-8w	Yes	8±1	Yes	Yes	Yes	Yes
EG08	RT-12w	Yes	12±1	Yes	Yes	Yes	Yes
EG09	RT-12w	Yes	12±1	Yes	Yes	Yes	Yes
EG10	RT-4w	Yes	4±1	Yes	No	Yes	No
EG11	RT-8w	Yes	8±1	Yes	No	Yes	No
EG12	RT-8w	Yes	8±1	Yes	No	Yes	No
EG13	RT-12w	Yes	12±1	Yes	No	Yes	No
EG14	RT-12w	Yes	12±1	Yes	No	Yes	No

*We excluded intravenous chemotherapeutic agents due to their potential confounding effects on gut microbiota and systemic inflammation. However, oral capecitabine was not excluded because it is an integral component of standard neoadjuvant chemoradiotherapy for locally advanced rectal cancer. All patients in the radiotherapy group received concurrent oral capecitabine as part of standard neoadjuvant chemoradiotherapy. This was consistently applied across all radiotherapy subgroups, minimizing within-group variability.

Of the 23 patients enrolled for inflammatory marker analysis, 18 patients (9 control, 9 radiotherapy) provided paired intestinal mucosal and saliva samples of sufficient quality for 16S rRNA gene sequencing. The remaining five patients (EG10, EG11, EG12, EG13, EG14; all from the radiotherapy group) were excluded from microbiome analysis due to poor saliva sample quality (e.g., visible blood contamination from gingival bleeding during mouth rinsing, low DNA yield, or insufficient sample volume). Two additional control patients (CG08, CG09) were included exclusively in the microbiome cohort; their intestinal mucosal inflammatory marker data were

subsequently measured and incorporated into the updated statistical analyses.
The final microbiome analysis cohort consisted of:
Control group (n=9): CG01–CG09
Radiotherapy group (n=9): EG01–EG09, comprising 4 patients from the RT-4w subgroup, 3 from the RT-8w subgroup, and 2 from the RT-12w subgroup.
